# TiO_2_/LaFeO_3_ Composites for the Efficient Degradation of Benzoic Acid and Hydrogen Production

**DOI:** 10.3390/molecules30071526

**Published:** 2025-03-29

**Authors:** Isabella Natali Sora, Benedetta Bertolotti, Renato Pelosato, Andrea Lucotti, Matteo Tommasini, Marica Muscetta

**Affiliations:** 1 Dipartimento di Ingegneria e Scienze Applicate, Università di Bergamo, Viale Marconi 5, 24044 Dalmine, Italy; benedetta.bertolotti@unibg.it (B.B.); renato.pelosato@unibg.it (R.P.); 2 Dipartimento di Chimica, Materiali e Ingegneria Chimica “G. Natta”, Politecnico di Milano, Piazza Leonardo da Vinci 32, 20133 Milano, Italy; andrea.lucotti@polimi.it (A.L.); matteo.tommasini@polimi.it (M.T.); 3 Dipartimento di Ingegneria Chimica, dei Materiali e della Produzione (DICMaPI), Università di Napoli Federico II, Piazzale V. Tecchio 80, 80125 Naples, Italy; marica.muscetta@unina.it

**Keywords:** lanthanum ferrite, benzoic acid degradation, photocatalytic hydrogen evolution, semiconductor photocatalyst

## Abstract

LaFeO_3_/TiO_2_ composites were prepared in the range 0–12.2 wt% of LaFeO_3_, characterized, and tested for both benzoic acid (BA) and 4-methoxycinnamic acid (MCA) degradation in aqueous solution, and hydrogen evolution. The preparation method was via ball-milling without thermal treatment. The composite materials presented agglomerates of LaFeO_3_ with an average size from 1 to 5 μm, and the TiO_2_ powder was well dispersed onto the surface of each sample. They showed varying activities for BA degradation depending on composition and light wavelength. The 6.2 wt% and 12.2 wt%-LaFeO_3_/TiO_2_ composites exhibited the highest activity under 380–800 nm light and could degrade BA in 300 min at BA concentration 13.4 mg L^−1^ and catalyst 0.12 g L^−1^. Using a 450 nm LED light source, all composites degraded less than 10% of BA, but in the presence of H_2_O_2_ (1 mM) the photocatalytic activity was as high as 96% in <120 min, 6.2 wt%-LaFeO_3_/TiO_2_ composite being the most efficient sample. It was found that in the presence of H_2_O_2_, BA degradation followed first order kinetic with a reaction rate constant of 4.8 × 10^−4^ s^−1^. The hydrogen production rate followed a classical volcano-like behavior, with the highest reactivity (1600 μmol h^−1^g^−1^ at 60 °C) in the presence of 3.86%wt- LaFeO_3_/TiO_2_. It was also found that LaFeO_3_/TiO_2_ exhibited high stability in four recycled tests without losing activity for hydrogen production. Furthermore, a discussion on photogenerated charge-carrier transfer mechanism is briefly provided, focusing on lacking significant photocatalytic activity under 450 nm light, so p-n heterojunction formation is unlikely.

## 1. Introduction

Many semiconductor oxides can act as photocatalysts [1,2,3,4,5]. Their activation occurs by irradiation with photons of energy that match the band gap energy of the semiconductor, resulting in electron excitation from the valence band (VB) to the conduction band (CB), thereby generating holes in the VB. Due to band bending, the photogenerated $e^{-}$-$h^{+}$ pairs diffuse to the semiconductor surface where, in the absence of electron and hole scavengers, their recombination takes place in a few nanoseconds. Recombination is avoided if an adequate scavenger or surface defect states are available to trap electron-hole pairs. In this case, water adsorbed or hydroxyl groups present on the semiconductor surface can be oxidized by $h^{+}$, generating $HO^{∙}\;$($H_{2}O+h^{+}\;\to \;{HO}^{∙}+H^{+}$), while adsorbed $O_{2\;ads}$ can be reduced by $e^{-}$, generating superoxide radicals $O_{2}^{∙-}$ ($O_{2\;ads}+e^{-}\;\to \;O_{2}^{∙-}$) [6]. The photocatalytic processes exploit the produced radicals to start a chemical reaction or to increase the reaction rate.

The broad utilization of semiconductor photocatalysts for organic pollutant decomposition in wastewater treatment plants has been limited by the high recombination of the photogenerated electron–hole pairs, resulting in low quantum efficiency of photocatalyzed reactions. Titanium dioxide (TiO_2_) is one of the most employed materials among photocatalysts [7,8]. It shows high activity when exposed to light and excellent chemical stability. Anatase TiO_2_ has an indirect and relatively large bandgap of E_g_ = 3.2 eV; for activation it requires the use of UV light which is more expensive and dangerous than radiation in the visible range [9]. Because of these challenges, nanocomposite catalysts in which one component consists of anatase TiO_2_ have been extensively studied with two main objectives: to improve the separation of photogenerated electron and hole pairs, and to obtain a TiO_2_-based photocatalyst also active under visible light irradiation.

In recent years, different types of materials (e.g., ceramic, metallic, carbon-based, and polymeric nanomaterials) have been combined with TiO_2_ to form functional nanocomposites, exhibiting attractive chemical and physical properties as compared to any of the components separately. LaFeO_3_ is a stable and non-toxic semiconductor with a p-type low band gap of 2.4–2.5 eV [10]. The use of LaFeO_3_ in photocatalytic degradation of persistent organic pollutants in wastewater, i.e., pharmaceuticals, has been previously investigated by us [11,12,13].

Although there are studies showing the visible-light photocatalytic degradation of (i) dyes by LaFeO_3_/TiO_2_ nanocomposite [14] and anatase-TiO_2_/rutile-TiO_2_/LaFeO_3_ three-phase nanostructured heterojunctions [15]; (ii) phenol by LaFeO_3_/TiO_2_ nanocomposite [16]; (iii) antibiotics by LaFeO_3_/TiO_2_ heterostructures [17] and by carbon quantum dot modified TiO_2_@LaFeO_3_ hollow core shell [18]; and (iv) pesticides by LaFeO_3_/TiO_2_ heterojunction [19] and core shell [20], as far as we know the photocatalytic degradation of aromatic carboxylic acids such as benzoic acid using LaFeO_3_/TiO_2_ has never been reported previously. Hydrothermal or wet chemical synthesis are the most common strategies for the preparation of these composites [14,16].

This study is focused on the photocatalytic performance of LaFeO_3_/TiO_2_ nanocomposites, (with 0–12.2 wt% of LaFeO_3_) prepared via a fast and simple ball-milling method without thermal treatment. Benzoic acid (BA) was chosen as a model compound suitable for investigating the photocatalytic behavior of aromatic acids in an aqueous environment. The photocatalytic activity was evaluated for the degradation of BA by UV-vis spectrophotometry and high-pressure liquid chromatography (HPLC). The photocatalytic activity of the composites was evaluated with and without hydrogen peroxide addition. Presently, H_2_ is mainly produced by steam methane reforming, which involves the use of fossil fuels and high temperature, resulting in low-purity hydrogen products and greenhouse gas emissions. Alternative more sustainable processes to produce high-purity H_2_ are via electrochemical and photochemical water splitting [21,22]. For better evaluation of the nanocomposites, the photocatalytic hydrogen production rate using methanol as a sacrificial agent was studied. The photocatalytic hydrogen evolution performance of LaFeO_3_/TiO_2_ has been the subject of a few investigations. Lv et al. [23] synthesized 20 wt%-LaFeO_3_/TiO_2_ nanocomposites by a 2-step approach and reported that they exhibited a H_2_ production rate of 0.279 mmol h^−1^ g^−1^ under the simulated solar light irradiation of a 300 W Xe lamp (320–780 nm). Comparison of photocatalytic water splitting H_2_ evolution rate of 20 wt%-LaFeO_3_/TiO_2_ without using methanol as a sacrificial agent gives 0.0178 mmol h^−1^ g^−1^ and no hydrogen generation was observed in the presence of bare TiO_2_ or LaFeO_3_. Recently, a 49.64 wt%-LaFeO_3_/TiO_2_ powder was prepared by Jiang et al. [24] via solid state method, annealing the mixture at 500 °C for 12 h in Ar atmosphere. The powder showed a high photocatalytic H_2_ production rate of ~3.26 mmol h^−1^ g^−1^ under AM 1.5 simulated sunlight, which is approximately 2.28 times that of commercial TiO_2_ P25 (1.43 mmol h^−1^ g^−1^).

## 2. Results and Discussion

### 2.1. Phase Composition and Microstructure

**Figure 1:**
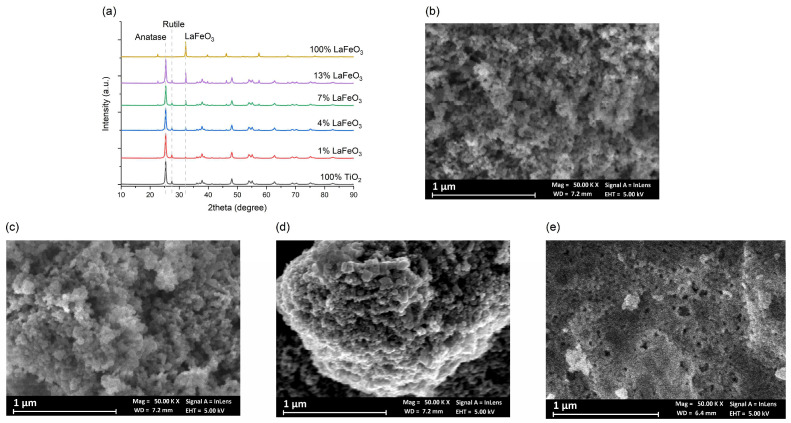
(**a**) XRD patterns of B-D-E nanocomposites and the reference compounds TiO_2_ and LaFeO_3_; (**b**) SEM image of C; (**c**) SEM image of D; (**d**) SEM image of E; (**e**) SEM image of LaFeO_3_.

As shown from X-ray diffraction (XRD) data, the LaFeO_3_ powder (sample F) is monophasic, and the diffraction peaks can be indexed with orthorhombic ABO_3_-type perovskite structure (JCPDS file No. 37-1493). The broad peaks suggest that obtained products have nanometric size. The XRD patterns of the composites B (1.3 wt%-LaFeO_3_/TiO_2_), C (3.9 wt%-LaFeO_3_/TiO_2_), D (6.2 wt%-LaFeO_3_/TiO_2_) and E (12.2 wt%-LaFeO_3_/TiO_2_) are presented in Figure 1a. Diffraction peaks belonging to the LaFeO_3_, TiO_2_ anatase (JCPDS card no. 21-1272), and TiO_2_ rutile (JCPDS card no. 21-1276) phases were detected in all composites. By increasing the LaFeO_3_ content (i.e., from B to E), the intensity of the orthoferrite diffraction peaks increases. No reaction products were observed in the XRD patterns of the composites. The XRD patterns confirm that the composites are physical mixtures of the two components as expected due to the short milling time. SEM micrographs of C, D and E composites are shown in Figure 1b–d. For brevity, other micrographs of the samples are shown in the Supporting Information (Figures S1 and S2). As shown in Figure S1, LaFeO_3_ powder is nanometric, very porous, and tends to agglomerate to larger particles, causing fast charge recombination. In the SEM micrographs of C, D and E, the same microstructure is evidenced: the agglomerates of LaFeO_3_ have an average size in the 1 to 5 μm range and the TiO_2_ powder is well dispersed onto the surface of each sample. This characteristic may have some relationship with their good performance on the photocatalytic degradation of benzoic acid.

To investigate the optical absorption characteristics of LaFeO_3_, nanoparticles at different wavelengths diffuse reflectance spectroscopy (DRS) analytical techniques were used. The UV–vis DRS spectrum (Figure S3) of LaFeO_3_ nanoparticles shows the absorption edge occurs at about 600 nm, attributed to its electron transitions from valence band (mainly mixed *e_g_* states of Fe *3d* and O *2p*) to conduction band (*t_2g_* states of Fe *3d*) [25]. A broad absorption band ranging from 200 to 600 nm indicates its potential applications as visible light-driven photocatalyst. LaFeO_3_ optical band-gap energy was 2.47 eV.

### 2.2. Benzoic Acid Degradation Using Daylight Lamps in LaFeO_3_/TiO_2_ Water Suspensions

**Figure 2:**
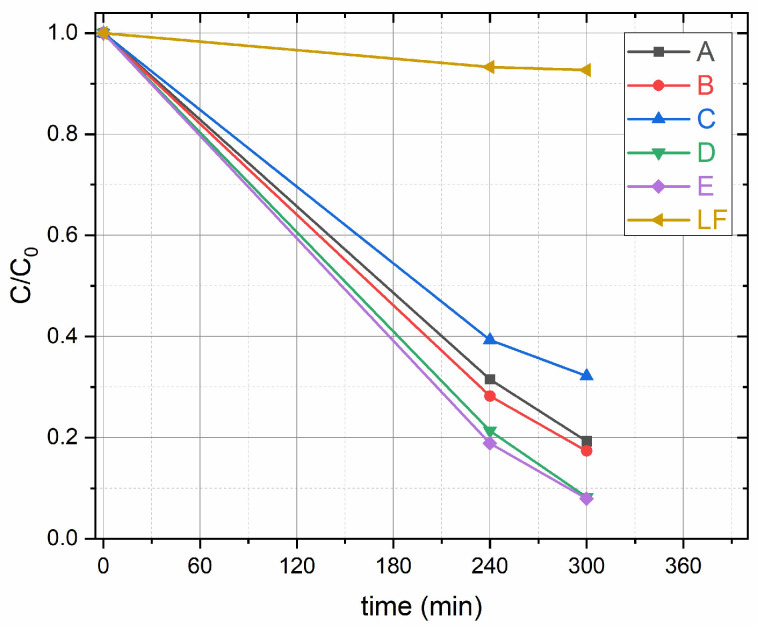
Photocatalytic degradation of BA in the presence of samples A (TiO_2_), B (1.3 wt%-LaFeO_3_/TiO_2_), C (3.9 wt%-LaFeO_3_/TiO_2_), D (6.2 wt%-LaFeO_3_/TiO_2_), E (12.2 wt%-LaFeO_3_/TiO_2_) and LF (LaFeO_3_) from HPLC analysis. Experimental conditions: 0.120 g L^−1^ catalyst loading, [BA] = 1 × 10^−4^ M, λ = 380–800 nm light.

Figure 2 shows the variation of the concentration of BA during the reactions over different catalysts, without an oxidizing agent such as H_2_O_2_, under visible light and a fraction of UV-A (380–800 nm). According to HPLC data, the LaFeO_3_/TiO_2_ composites degrade up to 93% of BA in 300 min. Pure TiO_2_ (sample A) and LaFeO_3_ (sample F) degraded 80% and 9% in 300 min, respectively. In Table 1 these results are compared with those of other LaFeO_3_/TiO_2_ photocatalysts on organic pollutant degradation using UV or visible light. The experimental studies are performed with a catalyst concentration in the range 0.8–2.4 g L^−1^, larger than the one used in this study (0.12 g L^−1^).

TiO_2_ powder covers LaFeO_3_ agglomerates, so it is likely that the BA adsorption on composites is similar to that of TiO_2_. BA adsorption depends on the surface charge of TiO_2_ and on the extent of BA deprotonation (*K*_a_ = 6.46 × 10^−5^ at 25 °C). The TiO_2_ surface is positively charged at pH below the point of zero charge at pH_zpc_ ≈ 6 [26]. At natural pH, pH ≈ 4.2–4.6 in A to E suspensions, BA adsorption should be favored through electrostatic interactions between the negatively charged benzoate ion C_6_H_5_COO^−^ in the water matrix and the –OH_2_^+^ groups on the TiO_2_ surface. In suspension F, pH is ≈6.1, and the benzoate would be attracted to the most positively charged LaFeO_3_ surface (pH_zpc_ = 8.9) through electrostatic interactions. It seems that no direct relationship can be established between the pH_zpc_ values and the photoactivity of the samples. However, given the good performance of the D and E composites in the degradation of BA, it could be assumed that BA is more easily degraded under a relatively weak acidic pH_zpc_ value, since it is a weak acid.

Another factor that could influence photocatalytic activity is the surface area of the catalysts. Perovskite oxides usually have small surface areas and large particle sizes. In particular, sample F (LaFeO_3_) presents a specific surface area of 14 m^2^ g^−1^ significantly smaller than that of bare TiO_2_ (48 m^2^ g^−1^), which is detrimental to the number of the available surface-active sites. Since the effective separation of the photogenerated $e^{-}$-$h^{+}$ pairs are a key aspect in photocatalytic reactions, the large particle size of LaFeO_3_ can have some relationship with a difficult migration of electrons or holes onto the surface of LaFeO_3_ to react with adsorbed BA rather than to recombine with each other. According to the mechanism described above, it is not difficult to understand that TiO_2_-containing composites show a higher photocatalytic activity compared to pure LaFeO_3_.

**Figure 3:**
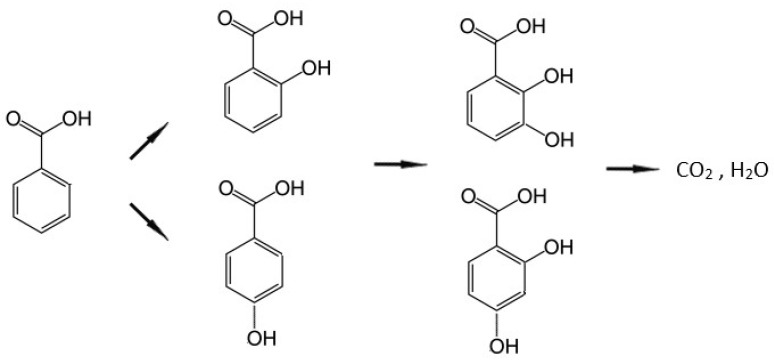
Pathway of degradation of benzoic acid following ref. [28].

As reported in previous studies [27] (Ajmera et al., 2002 [28]), the photocatalytic degradation of BA proceeds through HO^•^ radical attack preferentially on the *ortho* and *para* positions, producing hydroxy-benzoic acids and dihydroxy-benzoic acids, according to HPLC analysis. The intermediate species go through photocatalytic degradation in competition with BA, slowing down the BA degradation. As shown in Figure 3, a second hydroxylation and subsequent ring opening is likely to follow but the species expected to evolve from here would be small molecule organics (i.e., methanol) that would rapidly degrade to carbon dioxide.

**Figure 4:**
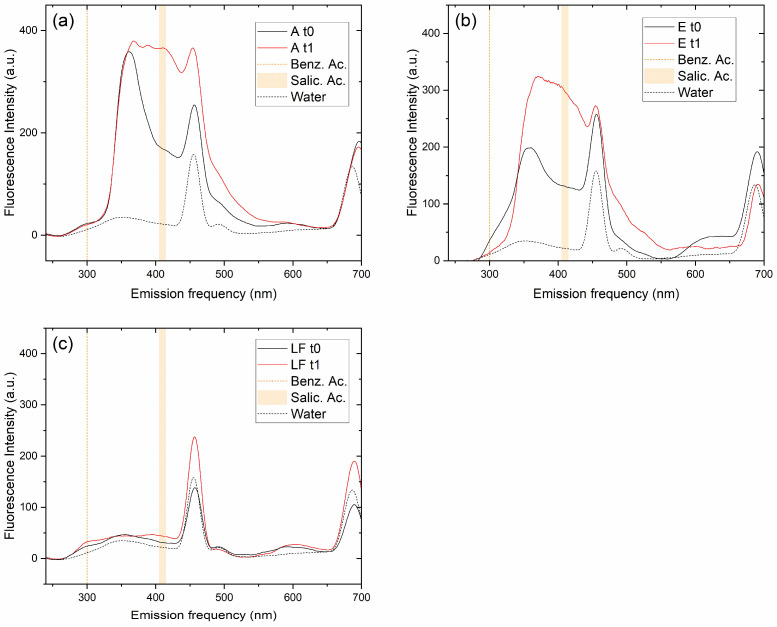
Fluorescence spectral patterns against UV irradiation time (t1 = 1 h) in the decomposition of benzoic acid in the presence of (**a**) sample A (TiO_2_), (**b**) sample E, and (**c**) sample F (LaFeO_3_). The excitation wavelength was 230 nm. Experimental conditions: 0.120 g L^−1^ catalyst loading, [BA] = 1 × 10^−4^ M, λ = 380–800 nm radiation. The measured solutions were obtained from the supernatant after centrifugation of the corresponding suspensions containing the photocatalyst. The peaks at ca. 460 nm and 690 nm are instrumental background, as evidenced by the spectrum of pure water excited in the same conditions, which is reported in each panel (^…..^ line) to guide the eye. The expected location of the signals from benzoic and salicylic acids is indicated by yellow lines.

The decrease of benzoic acid and the formation of salicylic acid after one hour of irradiation was assessed qualitatively using fluorescence spectroscopy. Figure 4 illustrates the temporal changes in the emission spectra of benzoic acid in aqueous solutions irradiated for one hour in the presence of a fixed amount of the photocatalysts A (Figure 4a), E (Figure 4b), and F (Figure 4c). Under identical conditions, benzoic acid emits significantly less than salicylic acid [29]. The small emission peak of benzoic acid at approximately 300 nm decreases after one hour of irradiation in the solutions prepared with A and E but not the one prepared with F. This is consistent with the HPLC data shown in Figure 2. For the photocatalysts A and E, after one hour of illumination, a new emission band appears at about 410 nm, which is a typical feature of salicylic acid excited at a wavelength of 230 nm [29]. Therefore, it is clear that salicylic acid is one of the intermediates produced during the degradation of benzoic acid by OH radical attack.

To assess the generality of the enhanced activity of the LaFeO_3_/TiO_2_ catalysts, the degradation of another aromatic carboxylic acid compound, namely 4-methoxycinnamic acid (MCA), was studied under previous experimental conditions. MCA undergoes to rapid and reversible trans-cis photoisomerization under UV radiation [30]. As shown in Figure S4, up to 98% of MCA is degraded in the presence of composite catalysts after 120 min of irradiation, better than pure TiO_2_ catalyst. To degrade 90% of the aromatic carboxylic acids the 6.2 wt% and 12.2 wt%-LaFeO_3_/TiO_2_ composites take <120 min in the case of MCA and 300 min for BA. Also, sample F shows a better performance with MCA substrate (72% in 120 min) as compared to BA (<10% in 300 min). As expected, the degradation of MCA is much faster than that of BA due to different degradation mechanisms. It is well known that the principal degradation pathway for cinnamic acid involves attack at the alkene group by a super oxide radical, generating benzaldehyde as the main intermediate. Benzaldehyde undergoes attack by hydroxyl radicals, generating OH substituted intermediates that are quickly decomposed to CO_2_ [31].

### 2.3. Benzoic Acid Degradation Under 450 nm Light in LaFeO_3_/TiO_2_ Water Suspensions With and Without H_2_O_2_

**Figure 5:**
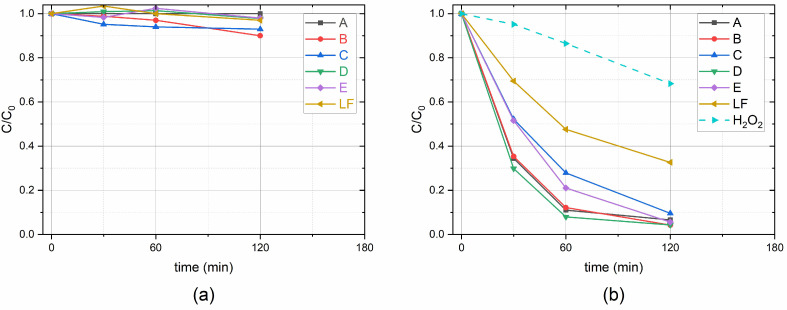
Photocatalytic degradation of BA in the presence of samples A (TiO_2_), B (1.3 wt%-LaFeO_3_/TiO_2_), C (3.9 wt%-LaFeO_3_/TiO_2_), D (6.2 wt%-LaFeO_3_/TiO_2_), E (12.2 wt%-LaFeO_3_/TiO_2_) and LF (LaFeO_3_), without H_2_O_2_ (**a**) and with H_2_O_2_ (**b**). Data from HPLC analysis. Experimental conditions: 0.120 g L^−1^ catalyst loading, [BA] = 1 × 10^−4^ M, [H_2_O_2_] = 1 mM, under monochromatic 450 nm light. For reference, the degradation of BA in the presence of H_2_O_2_ without catalyst is shown (blue dotted line).

The photocatalytic degradation of BA under monochromatic 450 nm light over different catalysts, with and without H_2_O_2_, was evaluated, and the results are shown in Figure 5. The results evidence that without H_2_O_2_ the bare photocatalysts and all LaFeO_3_/TiO_2_ composites degraded less than 10% of BA (Figure 5a). As shown in Table 1, Garcia-Muñoz et al. also reported a far smaller activity of the composite photocatalysts under >420 nm when compared to the reactions performed under solar light [19]. On the contrary, tests with dyes show high photocatalytic activity of LaFeO_3_/TiO_2_ composites under visible light [14]. However, tests with dyes (or with any substrate absorbing radiation) do not distinguish between a pure photocatalytic process and a dye-sensitized one, the latter being dye-specific and not general [32]. This could be the reason why pure LaFeO_3_ and LaFeO_3_/TiO_2_ composites, while demonstrated to work well in previous studies using dyes, were actually poorly effective without H_2_O_2_, as shown in the present work.

In the presence of H_2_O_2_ (1 mM), photocatalytic activity was high, and the most efficient samples were D, B and A. The pseudo-first-order rate constants (*k*) for BA degradation were determined for the initial reaction period (<120 min) and the values are reported in Table 2. The D composite shows the highest reaction rate constant (4.8 × 10^−4^ s^−1^), which is 1.2 and 3.2 times faster than those of TiO_2_ (4.1 × 10^−4^ s^−1^) and LaFeO_3_ (1.5 × 10^−4^ s^−1^), respectively. The radical intermediate HO^•^ formed from H_2_O_2_ by reaction with the photogenerated electrons can act as an electron scavenger, thus inhibiting the e^−^-h^+^ pairs recombination at the semiconductor surface [33] according to the following equation:

H_2_O_2_ + e_CB_^−^ → HO^•^ + OH^−^ (1)

where e_CB_^−^ indicates electron excited to the conduction band.

To further the study of the scarce activity of all photocatalysts without H_2_O_2_ under 450 nm light, degradation runs of 22 h were carried out. The results indicate that depending on composition, 30–60% of BA is degraded by the LaFeO_3_/TiO_2_ nanocomposites.

### 2.4. Hydrogen Evolution

Preliminary tests were performed by comparing the hydrogen evolution rate (HER) under UV-visible light radiation in the presence of pure TiO_2_ (P25), pure LaFeO_3_ and the composite materials B (1.3 wt%-LaFeO_3_/TiO_2_), C (3.9 wt%-LaFeO_3_/TiO_2_), D (6.2 wt%-LaFeO_3_/TiO_2_) and E (12.2 wt%-LaFeO_3_/TiO_2_), prepared by manual mixing of the two powders. Specifically, no hydrogen generation was observed in the presence of pure LaFeO_3_, due to the unsuitable band position of this photocatalyst (see Section 2.5), while the hydrogen amount was increased by 25% in the presence of the composite material when 1%wt. of LaFeO_3_ was used. Based on these results, it was chosen to evaluate the effect of LaFeO_3_ on HER, in the presence of methanol as the sacrificial agent.

**Figure 6:**
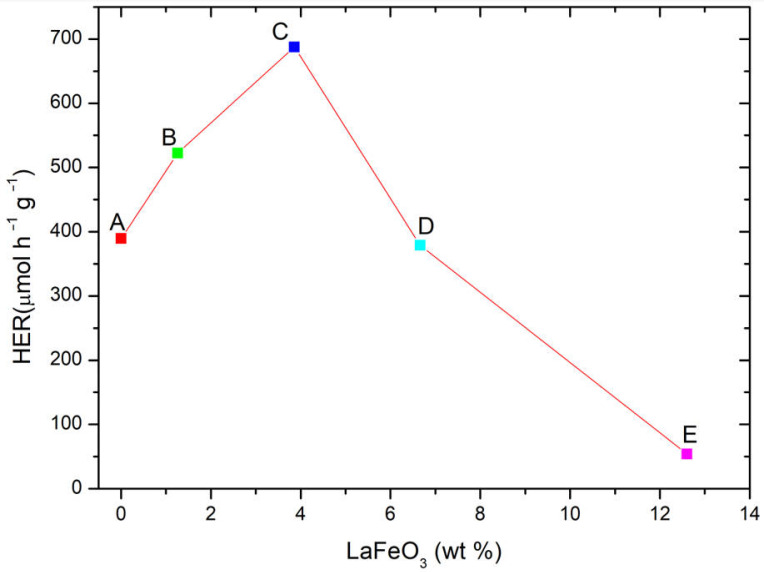
Photocatalytic hydrogen evolution rate (HER) at varying LaFeO_3_ (%wt.) with respect to P25. Methanol was used as a model scavenger. Experimental conditions: [Methanol] = 2.5 M; 0.5 g L^−1^ catalyst loading (Sample C); V = 0.3 L; T = 35 °C; natural pH of the solution.

As indicated in Figure 6, the hydrogen production rate follows a classical volcano-like behavior, with the highest reactivity in the presence of 3.86%wt of LaFeO_3_ (sample C). Beyond this value, the reduction of HER can be probably be ascribed to the excessive coverage of titanium dioxide.

**Figure 7:**
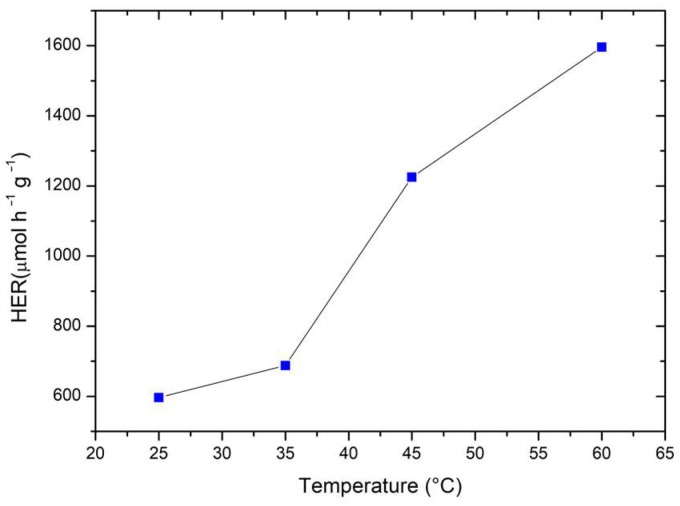
Photocatalytic hydrogen evolution rate (HER) at varying temperature of the system. Methanol was used as a model scavenger. Experimental conditions: [Methanol] = 2.5 M; 0.50 g L^−1^ catalyst loading (Sample C); V = 0.3 L; natural pH of the solution.

Furthermore, these materials were tested to assess the photocatalytic activity under visible light radiation. In this case, no hydrogen generation was observed probably due to (i) the wide band gap of titanium dioxide, as well as (ii) the unsuitable band gap position of LaFeO_3_ (see the next section). Some experiments were conducted to assess how the system’s temperature impacts photocatalytic hydrogen production. Indeed, temperature does not directly affect the photocatalytic process, as it primarily depends on the radiation wavelength and the material’s absorption properties. However, the system’s temperature may influence photocatalytic hydrogen generation by altering the adsorption behavior of organic species on the catalyst surface and modifying the reaction kinetics [34]. As illustrated in Figure 7, the amount of hydrogen produced increases as the system’s temperature rises with a HER 4.5-fold higher at the maximum tested temperature (i.e., 60 °C), with respect to the lowest temperature (i.e., 25 °C).

**Figure 8:**
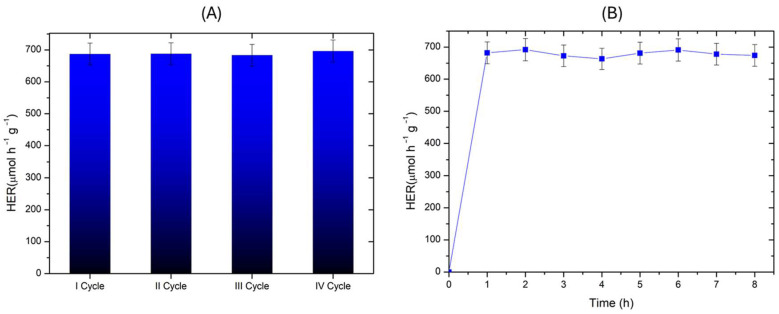
(**A**) Photocatalytic hydrogen evolution rate (HER) in successive photocatalytic tests. (**B**) Photocatalytic hydrogen evolution rate (HER) during a single photostability test. Methanol was used as a model scavenger. Experimental conditions: [Methanol] = 2.5 M; 0.50 g L^−1^ catalyst loading (Sample C); V = 0.3 L; natural pH of the solution; T = 35 °C.

Finally, photostability and reusability tests (Figure 8A,B) demonstrated good photostability both (i) during a single experiment carried out up to 8 h of irradiation, and (ii) during 4 photocatalytic cycles.

### 2.5. Activation of LaFeO_3_/TiO_2_ Nanocomposites

**Figure 9:**
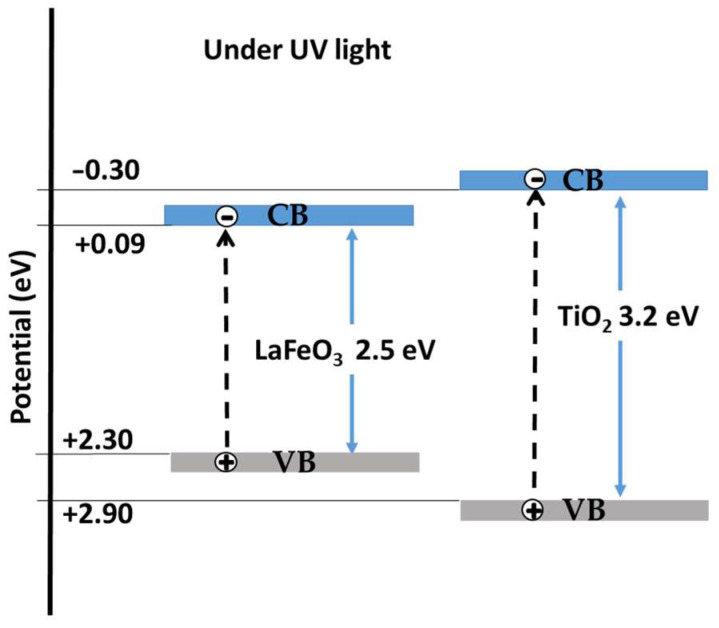
Scheme of the energy band structures and the correspondingly transfer process of photogenerated electron–hole pairs of semiconductor photocatalysts under UV light.

Different types of photogenerated charge-carrier transfer mechanism have been proposed for LaFeO_3_/TiO_2_ composites: type-II double charge transfer mechanism [14], direct Z-scheme mechanism [23], S-scheme [20], and uncommon heterojunction [16]. The formation of a p–n heterojunction between TiO_2_ (n-type semiconductor) and LaFeO_3_ (p-type semiconductor) could avoid $e^{-}$-$h^{+}$ recombination and improve the activity. The valence band potential of a semiconductor at the point of zero charge can be calculated by the following empirical equation:

E_VB_ = X − E^e^ + 0.5E_g_ (2)

where E_VB_ is the VB edge potential, Χ is the electronegativity of the semiconductor, which is the geometric mean of the electronegativity of the constituent atoms, E^e^ is the energy of free electrons on the hydrogen scale (about 4.5 eV), and E_g_ is the band gap energy of the semiconductor [35]. The conduction band potential E_CB_ can be determined by the equation E_CB_ = E_VB_ − E_g_. The X values for LaFeO_3_ and TiO_2_ (anatase) are about 5.57 and 5.78 eV, respectively. The calculated E_CB_ and E_VB_ edge potentials for LaFeO_3_ are +0.1 eV and +2.3 eV vs. SHE, as shown in Figure 9. For TiO_2_, E_CB_ and E_VB_ are −0.3 eV and +2.9 eV vs. SHE, in agreement with previous studies [17,20]. Before contact between the semiconductors, the calculated E_VB_ of LaFeO_3_ is under that of the TiO_2_ and E_CB_ is above that of the TiO_2_. The creation of a p–n heterojunction would modify the electronic band structure of both semiconductors. Since composites do not have significant photocatalytic activity under visible light in these samples, p–n heterojunction formation is quite unlikely, so a high rate of recombination of photogenerated charge is present.

In our study, the influence of the LaFeO_3_ content was as follows: (i) under pure visible light better results are achieved by samples with a lower amount of LaFeO_3_ (B and C); (ii) under visible light and a fraction of UV-A radiation, samples with higher wt% of LaFeO_3_ (D and E) are more active; (iii) finally, the composites analyzed were not able at all to produce hydrogen under visible light. This behavior can suggest different light-mediated reaction mechanisms depending on the light wavelengths. Also, Garcia-Muñoz et al. reported the influence of the LaFeO_3_ content on degradation of myclobutanil, but the optimum was observed at 5 wt% under solar light and 12.5 wt% under visible light, respectively [19]. The different behavior it is probably related to the core-shell system study by Garcia-Muñoz et al., while our composites are physical mixtures of the two components where TiO_2_ particles are well dispersed on LaFeO_3_ agglomerates.

## 3. Materials and Methods

### 3.1. Chemicals and Preparations

Reactants for synthesis and chemicals for the photocatalytic reactions were purchased from Sigma-Aldrich (Saint Louis, MO, USA): La_2_O_3_, Fe(NO_3_)_3_·9H_2_O, citric acid hydrate (C_6_H_8_O_7_·xH_2_O, 99.5%), H_2_O_2_ (>30%), nitric acid and NH_3_ (30% aqueous solution). Lanthanum ferrite LaFeO_3_ nanopowders were prepared using the citrate auto-combustion method. A specific amount of dried La_2_O_3_ was dissolved in a nitric acid solution to prepare La(NO_3_)_3_∙6H_2_O. Stoichiometric amounts of metal nitrates were dissolved in water (0.1 mol L^−1^) by stirring on a hotplate. The solution was poured in citric acid solution (molar ratio of metal ions to citric was 1:1). Aqueous NH_3_ was added until the pH was 6.8. The solution was then dehydrated until a brown/orange gel formed. Dry gel was heated in air to 250 °C to start ignition. The powder was then calcined at 600 °C (5 °C min^−1^) in air for 3 h to decompose ammonium nitrate residues. A fast and simple procedure was employed to prepare the LaFeO_3_/TiO_2_ photocatalysts [36]. The method involved manual mixing of fixed quantities of TiO_2_ (P25, nanopowder, mean particle diameter <50 nm, Sigma Aldrich) and LaFeO_3_ in a vial. The wt% of LaFeO_3_ to TiO_2_ was 0 (sample A), 1.3 (sample B), 3.9 (sample C), 6.2 (sample D), 12.2 (sample E), and 100 (sample F) wt%. Then, a ball-milling method with an agate mortar and agate balls in a planetary mill was applied (PM100, RETSCH, Haan, Germany). The rotational speed (rpm) and milling time (min) were fixed at 1 min and 200 rpm, based on the previous studies on similar systems.

### 3.2. Microstructural Characterization

The powder X-ray diffraction patterns of the catalysts were recorded using a Bruker (Milano, Italy) D8-Advance powder diffractometer equipped with a Cu kα X-ray source and a Lynxeye XE-T^®^ solid-state detector. The patterns were recorded in an interval of 10–90° 2θ with a step of 0.01° and a counting time of 1 s per step. Brunauer–Emmett–Teller (BET) specific surface area (SSA) determination was performed with nitrogen absorption on about 500 mg of the samples using a Micrometric (Norcross, GA, USA) Tristar 3000 automated gas-adsorption analyzer.

Scanning electron microscopy (SEM) was performed using a FEI (Hillsboro, OR, USA) Quanta 200 ESEM microscope, operating at 20 kV on specimens from which a thin layer of gold had evaporated. On the other hand, an electron microprobe used in an energy dispersive mode (EDX) was employed to obtain information on the actual metal–content ratio present in the samples. The UV-Vis diffuse reflectance spectroscopy (DRS) spectrum of LaFeO_3_ powder was recorded in the 200–800 nm range by using a Jasco (Tokyo, Japan) V-650 spectrophotometer equipped with an integrating sphere for solid samples, with BaSO_4_ as the reference sample. The plot of the Kubelka–Munk function (F(R_∞_) × E)^2^ vs. photon energy (E) suggests a direct bandgap transition of 2.47 eV for LaFeO_3_

### 3.3. Florescence Spectroscopy

The fluorescence spectra reported in this work were recorded with a JASCO FP 6600 spectrofluorometer selecting the excitation wavelength of 230 nm, which corresponds to the experimental condition used to investigate photodegradation phenomena in benzoic/salicylic acids (Hidaka et al., 2006 [29]). The sample was placed in a Suprasil^®^ quartz cuvette and the emission signal was collected at 90° to the excitation beam.

### 3.4. Photocatalytic Studies

The photocatalytic activity was evaluated by the degradation of benzoic acid (BA) and 4-methoxycinnamic acid (MCA); their main characteristics are shown in Table 3. The tests were performed under UV (LED lamp, 365 nm) and visible light irradiation (LED lamp, 450 nm Penn Photoreactor M2 and Multiray Daylight/fluorescent 380–800 nm). In a typical test, 2.4 mg of photocatalyst powder was added to 20 mL of a 1.1 × 10^−4^ M (13.4 mg L^−1^) aqueous solution of BA. Before turning on the light, the mixture was stirred in the dark for 20 min in a 40 mL vial in order to allow adsorption/desorption equilibrium on the catalyst surface. At different time intervals (0 ÷ 5 h), aliquots of the reacting suspension (2.6 mL) were collected. The samples were centrifugated for 10 min at 450 rpm and the supernatants were analyzed with a UV-Vis spectrophotometry and high-pressure liquid chromatography, HPLC (Agilent 1200 series, CA, USA, column Zorbax Eclipse XDB-C18 4.6 × 150 mm, λ 228 nm, eluent water/methanol 6:4 + 1.5% acetic acid). The catalytic activity of the composites was evaluated with and without hydrogen peroxide addition (1 mM).

A 0.3 L glass batch reactor was employed for the photocatalytic hydrogen evolution experiments, equipped with a medium-pressure mercury lamp (Nominal Power P = 125 W) and a thermostat to control the temperature of the system. At a fixed temperature (25 °C ÷ 60 °C), during a typical experiment, a selected amount of photocatalyst (500 ppm) was suspended in a methanol aqueous solution (2.5 M). To prevent the reaction between dissolved oxygen and the photogenerated electrons, a nitrogen (N_2_) stream was bubbled into the solution starting 40 min before the experiment. A Gas Chromatograph (Agilent 7820A) with an HP-PLOT Molesieve 5A column (Agilent) and a TCD detector, with argon as the carrier gas was utilized for the estimation of hydrogen production.

## 4. Conclusions

We have developed a facile and cost-effective approach to the preparation of a LaFeO_3_/TiO_2_ (P25) composites with LaFeO_3_ wt% up to 12.2%. SEM analysis of the composites evidenced that the agglomerates of LaFeO_3_ have an average size in the 1 to 5 μm range and the TiO_2_ powder is well dispersed onto the surface of each sample. This characteristic seems to have some relationship with their good performance on the photocatalytic degradation of benzoic acid. The composites display enhanced photocatalytic activity when 380–800 nm light is used for irradiation; for example, 6.2 wt% and 12.2 wt%-LaFeO_3_/TiO_2_ composites degrade 93% of BA in 300 min compared to 80% for TiO_2_. Fluorescence measurements corroborated that salicylic acid is one of the intermediates formed during the degradation of BA by OH• radical attack. Under 450 nm LED light, all LaFeO_3_/TiO_2_ composites degraded less than 10% of BA, but in the presence of H_2_O_2_ (1 mM) the photocatalytic activity was as high as 96% in <120 min, 6.2 wt%-LaFeO_3_/TiO_2_ composite being the most efficient sample. Then, the catalysts were suspended in a methanol aqueous solution (2.5 M) and irradiated under UV-visible light. A hydrogen evolution rate of 700 μmol h^−1^g^−1^ was measured for the 3.86%wt-LaFeO_3_/TiO_2_ nanocomposite and good recyclability was established. The photocatalytic activity of LaFeO_3_/TiO_2_ composites is characterized by a volcano-like profile for both the BA degradation and the hydrogen evolution, but the optimum semiconductors ratio (LaFeO_3_:TiO_2_) depends on the wavelength of activating light. The mechanism of the photocatalytic degradation is proposed to involve the photoactivation of both LaFeO_3_ and TiO_2_ phases, leading to the formation of photogenerated charges ($e^{-}$-$h^{+}$) in both semiconductors. Further, the improvement of the photoactivity of the composites can be attributed to their larger surface area compared to pure LaFeO_3_ and good dispersion of TiO_2_ over the surface of LaFeO_3_, which might have decreased the recombination center.

## Figures and Tables

**Table 1:** Pollutant photodegradation over LaFeO_3_/TiO_2_ composites.

Photocatalyst	LaFeO_3_:TiO_2_	Radiation	Organic Compound	Catalyst	Removal (%)	Time (min)	Ref.
LaFeO_3_/TiO_2_ rutile calcination 800 °C/6 h	1.5:1 (m/m)	visible	methyl orange	n.a.	90	180	[14]
LaFeO_3_@TiO_2_ heterojunction	5:100	300–800 nm	myclobutanil 20 mg L^−1^	1 g L^−1^	100	180	[19]
LaFeO_3_@TiO_2_ heterojunction	12.5:100	>420 nm	myclobutanil 20 mg L^−1^	1 g L^−1^	37	240	[19]
LaFeO_3_/TiO_2_ anatase calcination 500 °C/3 h	3:100	>420 nm	ciprofloxacin 10 mg L^−1^	2.4 g L^−1^	100	90	[17]
LaFeO_3_/TiO_2_ (P25) calcination 450 °C/2 h	0.85:100	254 nm	thiamethozam 20 mg L^−1^	0.8 g L^−1^	97	120	[20]
LaFeO_3_/TiO_2_ (P25) calcination 450 °C/2 h	0.85:100	direct sunlight	thiamethozam 20 mg L^−1^	0.8 g L^-1^	79	120	[20]
LaFeO_3_/TiO_2_ (P25) ball-milling	6.2:100	LED450 nm	benzoic acid 13.4 mg L^−1^	0.12 g L^−1^ H_2_O_2_ 1 mM	96	120	this work
LaFeO_3_/TiO_2_ (P25) ball-milling	6.2:100	LED 450 nm	benzoic acid 13.4 mg L^−1^	0.12 g L^−1^	9	120	this work
LaFeO_3_/TiO_2_ (P25) ball-milling	6.2:100	380–800 nm	benzoic acid 13.4 mg L^−1^	0.12 g L^−1^	90	300	this work

**Table 2:** Values of the rate constant (*k*) for benzoic acid, and correlation coefficients of the linear fittings of the experimental data according to the pseudo-first-order reaction kinetic. Experimental conditions: 0.120 g L^−1^ catalyst loading, [BA] = 1 × 10^−4^ M, [H_2_O_2_] = 1 mM, monochromatic 450 nm light.

Catalyst	*k* (s^−1^)	R^2^
A	4.1 × 10^−4^	0.934
B	4.4 × 10^−4^	0.969
C	3.2 × 10^−4^	0.996
D	4.8 × 10^−4^	0.943
E	4.0 × 10^−4^	0.995
F	1.5 × 10^−4^	0.967

**Table 3:** Main physical–chemical properties of benzoic acid and 4-methoxycinnamic acid.

	Benzoic Acid	4-Methoxycinnamic Acid
N. CAS	65-85-0	943-89-5
Chemical structure	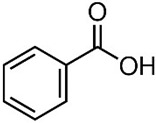	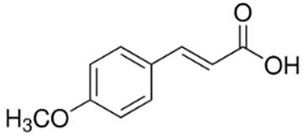
Molecular formula	C_6_H_5_COOH	C_10_H_10_O_3_
Molecular mass	122.12 g/mol	178.18 g/mol
Physical form	White powder	White powder
Solubility in water	3.4 g/L at 25 °C	0.71 g/L at 25 °C
pKa	4.19	4.60

## Data Availability

The data presented in this study are available on request from the corresponding author.

## Supplementary Materials

The following supporting information can be downloaded at https://www.mdpi.com/article/10.3390/molecules30071526/s1, Figure S1: SEM images of LaFeO3 powder. Figure S2: SEM image and EDX spectrum of C sample. Figure S3: Plot of Kubelka-Munk function (F(R∞)*E)2 vs. photon energy obtained from DRS for band gap calculation of LaFeO3. Figure S4: Photocatalytic degradation of MCA up to 120 min of irradiation from UV-vis data. Experimental conditions: 0.120 g L−1 catalyst loading, [MCA] = 2.5 × 10^−5^ M under 380–800 nm light. For reference, the MCA photolysis is reported (red stars).
