# Supplementary material for: TiO2/LaFeO3 Composites for the Efficient Degradation of Benzoic Acid and Hydrogen Production
Source: Molecules. 2025 Mar 29;30(7):1526. doi: 10.3390/molecules30071526 (PMC11990147; doi:10.3390/molecules30071526)
Supplement: Supplementary file 1 [file molecules-30-01526-s001.zip › molecules-3557364-supplementary.pdf]

molecules

Supplementary materials

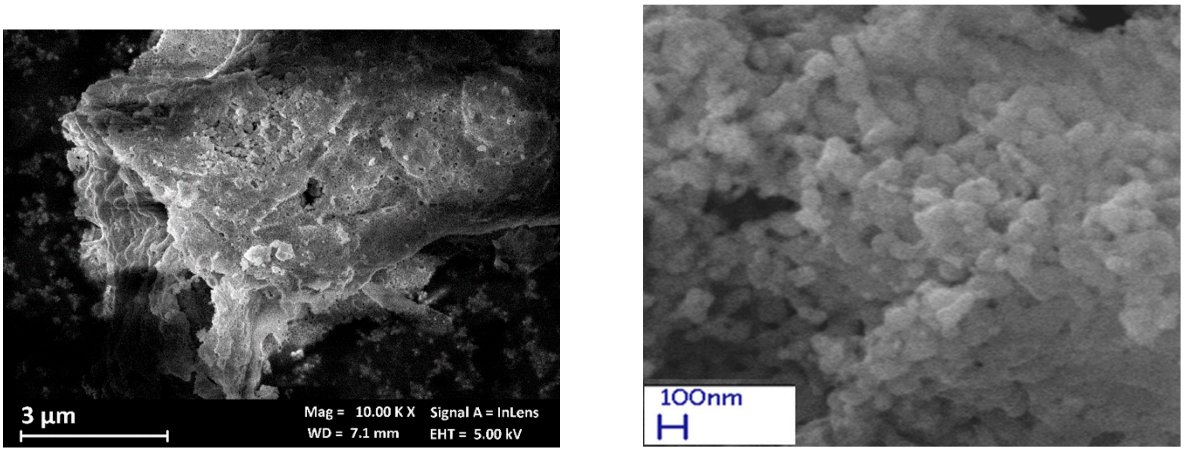

Figure S1: SEM images of $LaFeO_3$ powder.

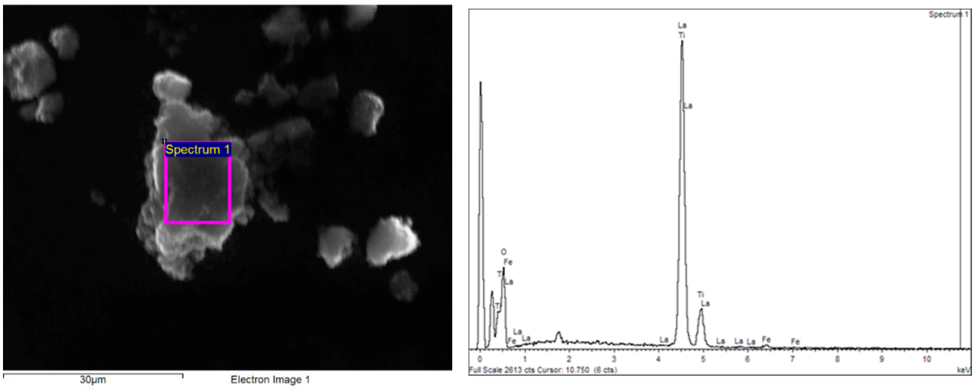

Figure S2: SEM image and EDX spectrum of C sample.

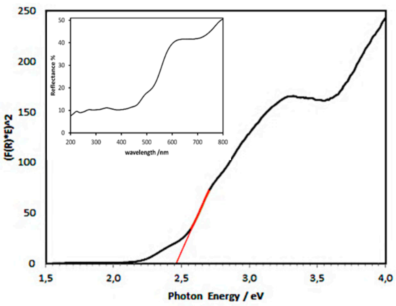

Figure S3: Plot of Kubelka-Munk function $(F(R_\infty)*E)^2$ vs. photon energy obtained from DRS for band gap calculation of $LaFeO_3$.

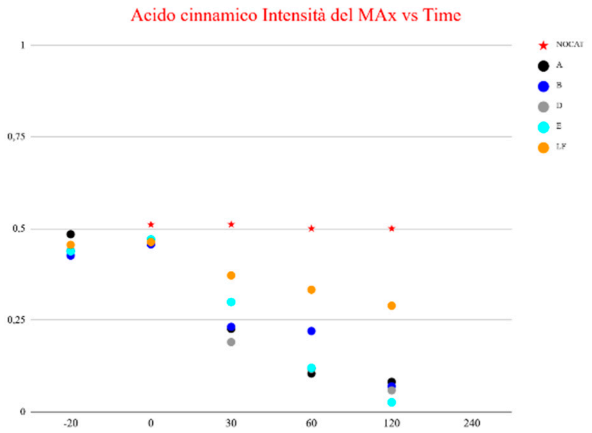

Figure S4 Photocatalytic degradation of MCA up to 120 min of irradiation from UV-vis data. Experimental conditions: 0.120 g L^{-1} catalyst loading, [MCA] = 2.5 x 10^{-5} M under 400-800 nm light. For reference, the MCA photolysis is reported (red stars).
